# Micrococcal Nuclease Does Not Substantially Bias Nucleosome Mapping

**DOI:** 10.1016/j.jmb.2012.01.043

**Published:** 2012-03-30

**Authors:** James Allan, Ross M. Fraser, Tom Owen-Hughes, David Keszenman-Pereyra

**Affiliations:** 1 Institute of Cell Biology, University of Edinburgh, Darwin Building, King's Buildings, West Mains Road, Edinburgh EH9 3JR, Scotland, UK; 2 Wellcome Trust Centre for Gene Regulation and Expression, School of Life Sciences, University of Dundee, Dundee DD1 5EH, Scotland, UK

**Keywords:** MNase, micrococcal nuclease, CAD, caspase-activated DNase, BLG, β-lactoglobulin, YRO, yeast replication origin, PDB, Protein Data Bank, caspase-activated DNase, nucleosome positioning, β-lactoglobulin, yeast replication origin, micrococcal nuclease

## Abstract

We have mapped sequence-directed nucleosome positioning on genomic DNA molecules using high-throughput sequencing. Chromatins, prepared by reconstitution with either chicken or frog histones, were separately digested to mononucleosomes using either micrococcal nuclease (MNase) or caspase-activated DNase (CAD). Both enzymes preferentially cleave internucleosomal (linker) DNA, although they do so by markedly different mechanisms. MNase has hitherto been very widely used to map nucleosomes, although concerns have been raised over its potential to introduce bias. Having identified the locations and quantified the strength of both the chicken or frog histone octamer binding sites on each DNA, the results obtained with the two enzymes were compared using a variety of criteria. Both enzymes displayed sequence specificity in their preferred cleavage sites, although the nature of this selectivity was distinct for the two enzymes. In addition, nucleosomes produced by CAD nuclease are 8–10 bp longer than those produced with MNase, with the CAD cleavage sites tending to be 4–5 bp further out from the nucleosomal dyad than the corresponding MNase cleavage sites. Despite these notable differences in cleavage behaviour, the two nucleases identified essentially equivalent patterns of nucleosome positioning sites on each of the DNAs tested, an observation that was independent of the histone type. These results indicate that biases in nucleosome positioning data collected using MNase are, under our conditions, not significant.

## Graphical Abstract

**Figure:**
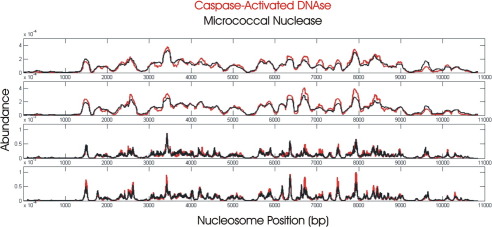

**Highlights:**

► We measured nucleosome positioning using two distinct nucleases. ► CAD and MNase provided equivalent positioning profiles. ► The results were independent of DNA and histone type used to prepare chromatin. ► Our data are not consistent with the proposal that MNase provides biased nucleosome positioning measurements.

## Introduction

Nucleosome positioning plays a fundamental role in determining chromatin structure and, consequently, in regulating genetic activity.^1–3^ Although an awareness of the capacity of histone octamers to adopt particular positions with respect to the underlying DNA was established many years ago, a detailed appreciation of the extent of its occurrence has only become available in recent years, mainly due to the implementation of second-generation sequencing technologies.^3^ Nevertheless, some of the controversies that have accompanied this topic since its inception continue to raise concerns.

Currently, the basis of the main approach used to map nucleosome positioning on genomic DNA involves fragmenting chromatin, native or reconstituted, to mononucleosomes and sequencing the DNA recovered from these structures.^3^ Although fragmentation is sometimes accomplished by sonication, after cross-linking, it is most frequently achieved by digesting the chromatin with micrococcal nuclease (MNase). Recently, the validity of using this enzyme for nucleosome positioning studies has, again, been questioned, and it has been suggested that results obtained with the probe may be biased^4–6^ and possibly artifactual.^5^ The foundation for this argument is the observation that when nucleosomal-length DNA fragments are isolated from a sample of protein-free DNA that has been digested with MNase, their sequences are correlated to both *in vivo* and *in vitro* nucleosome positioning sites mapped onto the same DNA. Thus, it is argued, nucleosome positioning data appear to simply reflect a cutting preference of MNase combined with a process of size selection.^5^

The problems associated with MNase are perceived to be twofold. Firstly, the enzyme displays notable sequence-specific cleavage with a preference to cut at sites centred on A/T-containing dinucleotides.^7–9^ This issue is a particular problem for indirect, end-labelling-based methods that map nucleosome positioning after very mild digestion with MNase, and in these studies, careful protein-free DNA controls are required.^10^ On the other hand, for chromatin extensively digested to mononucleosomes, as is consistently the case when positioning is assessed by DNA sequencing, this bias should not present a substantial concern in terms of identifying the histone-protected positioning sites. However, if the enzyme can attack the DNA that is wrapped around the histone octamer, rather than restricting cleavage to the linker DNA between nucleosomes, then, in combination with its sequence specific cleavage behaviour, the enzyme has the potential to selectively digest nucleosomes containing a high proportion of A/T-containing dinucleotides and effectively remove them from the population of DNA fragments destined for sequencing. This scenario and the implications for the quantitative identification of nucleosome positioning were initially perceived by McGhee and Felsenfeld in 1983.^11^

If the above problems relating to the use of MNase do impact substantially upon the identification of nucleosome positioning, it would have a number of implications concerning our understanding of the biological role of the occurrence and the extent to which it is determined by DNA sequence. Clearly, therefore, an assessment of the extent of the MNase bias must be established.

**Fig. 1:**
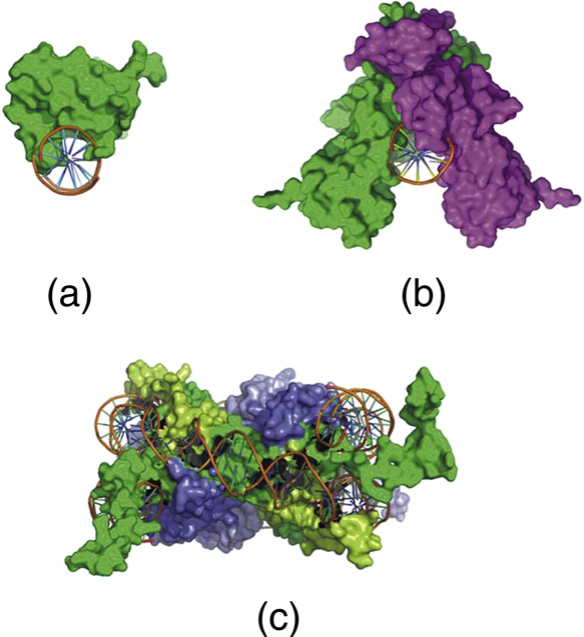
Schematic representation and comparison of the binding of (a) monomeric MNase^20^ (PDB ID: 2SNS) and (b) dimeric, caspase-activated DNase^17^ (PDB ID: 1V0D) to DNA during cleavage. The proposed structures of the complexes are hypothetical and are based on the high-resolution structures of the enzymes and the locations of their catalytic sites. For reference, the core particle structure^21^ (PDB ID: 1KX5), viewed along the nucleosomal dyad axis, is also shown (c).

Caspase-activated DNase (CAD) fragments genomic chromatin during apoptosis.^12–16^ The precursor for the enzyme is maintained in the nucleus as an inactive heterodimer containing the nuclease subunit (CAD/DFF40, hereafter termed CAD) and a chaperone (DFF45), which acts as an inhibitor.^16–18^ When, during the apoptotic cascade, the DFF45 inhibitor is cleaved by caspase-3, it releases the CAD nuclease, allowing the formation of homodimers that are the enzymatically active form of the enzyme.^16^ The crystal structure of CAD nuclease^17,19^ suggests that the dimer adopts a structure akin to a pair of scissors in which the active site is located deep within the crevice between the scissor blades (Fig. 1). This structure of CAD and the mechanism by which it cleaves double-stranded DNA is consistent with the preference for rotational symmetry in the sequence of the favoured cleavage site.^22,23^ It also explains the inability of the enzyme to cut DNA bound to the histone octamer and thus to be almost exclusively restricted in its action on chromatin to the cleavage of the linker DNA between nucleosomes,^23^ a feature particularly important in the context of the nucleosome mapping.

In the current study, we have compared sequence-directed, *in vitro* nucleosome positioning data derived from reconstituted chromatin digested with either MNase or CAD. As the results obtained with the latter probe are unlikely to be biased as a consequence of intranucleosomal cleavage,^23^ they should serve as a suitable reference to assess the extent of the potential bias introduced by MNase. The locations and relative affinities of the binding sites for both chicken and frog histone octamers on two separate genomic DNA molecules were determined by high-throughput sequencing of mononucleosomal DNA fragments produced using either of the two types of nucleases. By comparing these two data sets, we found no evidence to support the claim that nucleosome positioning data are substantially biased by the use of MNase.

## Results

### Preparation and properties of the nucleosome sequence data sets

A mixture of two plasmid DNAs, one harbouring a 10,841-bp segment of sheep DNA containing the β-lactoglobulin (BLG) gene and the other harbouring a 13,626-bp segment of *Saccharomyces cerevisiae* DNA incorporating a late-firing replication origin [yeast replication origin (YRO)] was reconstituted with limiting amounts of core histones by salt gradient dialysis.^24,25^ In separate reconstitutions, two different types of core histones were employed: chicken histones, isolated from mature erythrocytes, and recombinant frog histone octamers. The resulting chromatins were divided into two aliquots, one of which was digested with MNase and the other with CAD. Conditions were chosen to produce an equivalent extent of overall digestion and recovery of mononucleosomal DNA with the two enzymes (Supplementary Fig. 1). Mononucleosomal DNA fragments, recovered from the digested chromatin, were purified by gel electrophoresis. It was evident at this stage that monomer DNA recovered from CAD-digested chromatin was slightly longer than the equivalent samples prepared with MNase (Supplementary Fig. 1).

Using these DNA populations, we determined the locations and relative abundance of the preferred sites of histone octamer positioning on the different DNAs by high-throughput sequencing. Illumina/Solexa paired-end sequencing provided, on average, a total of 7.4 (± 0.4) × 10^6^ reads per sample, of which 97.6 (± 0.4)% uniquely aligned with high confidence to the reference sequences.

The number of reads that aligned to each of the two DNA sequences (excluding the plasmid vectors) was strongly biased with respect to the source of DNA. The number of reads mapping to the sheep BLG sequence was consistently greater than expected (observed-to-expected ratio of 1.38 ± 0.07), whereas the reads mapping to the YRO sequence were notably underrepresented (0.34 ± 0.06). As pairs of DNAs were reconstituted, digested, and gel purified as a mixture, the differences in read numbers cannot be attributed to variation in these aspects of the processing procedure. Given the competitive conditions used for reconstitution, the bias could well reflect the average base compositions of the two sequences (BLG and YRO: 56.0% and 38.6% GC, respectively) and the known preference of the histone octamer for GC-rich DNA.^26–29^

**Fig. 2:**
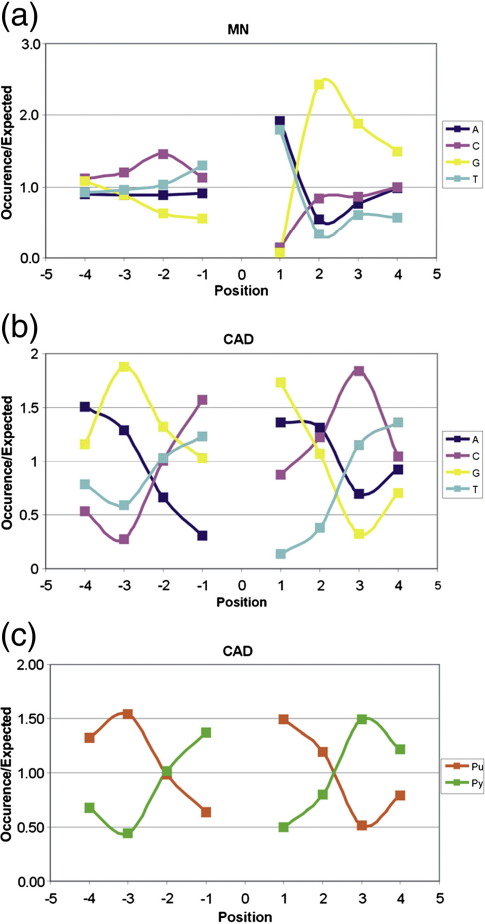
Sequence properties of the cleavage sites for MNase and caspase-activated DNase. The occurrence of each nucleotide, both 5′ and 3′ of the cleavage point (position 0), for binding sites identified with MNase (a) or CAD nuclease (b) is shown. In addition, for CAD nuclease, the results are presented in terms of the occurrence of purines and pyrimidines (c). These data are an average of all sites identified from sequence reads obtained from nucleosomal DNAs prepared with BLG and YRO reconstituted with either frog or chicken histones. Separate results for BLG and YRO alone are presented in Supplementary Fig. 2.

The preferences in cutting-site sequence for MNase and CAD on our chromatins were investigated by examining the abundance of each nucleotide in the sequence immediately surrounding the points of cleavage (Fig. 2; Supplementary Fig. 2). Both nucleases display some sequence specificity. For each enzyme, a similar bias was seen on both the AT-rich (YRO) and GC-rich (BLG) sequences (Supplementary Fig. 2). Consequently, the data presented in Fig. 2 are an average for both DNAs.

With MNase, it is most notable that there is an almost exclusive occurrence of an A or T immediately 3′ of the cleavage site and that this nucleotide is frequently followed by the dinucleotide GG (Fig. 2). At the 5′ side of the cut, a pronounced sequence preference is less evident. The overall pattern of preference in cleavage sequence for MNase is consistent with previous results.^8,30^ CAD nuclease has previously been shown to exhibit a strong preference to cut at sequences that display rotational (dyad) symmetry in the distribution of purines and pyrimidines and, consequently, a bias towards sequences of the type PuPuPuPy↓PuPyPyPy.^22,23^ In our data, the CAD cleavage site sequences exhibit striking rotational symmetry in terms of the distribution of purines and pyrimidines (Fig. 2) and indicate a preference to cut at PuPuNPy↓PuNPyPy (where N represents any nucleotide). These results are entirely consistent with the prior observation.^22^ Thus, in spite of the limitations of our analysis, in that it is focused on a limited amount of DNA sequence (compared, for example, to a whole genome study), both MNase and CAD clearly display bias in respect of the sites at which they prefer to cleave DNA, and in this context, the two enzymes appear quite distinct in terms of their particular sequence preferences.

**Fig. 3:**
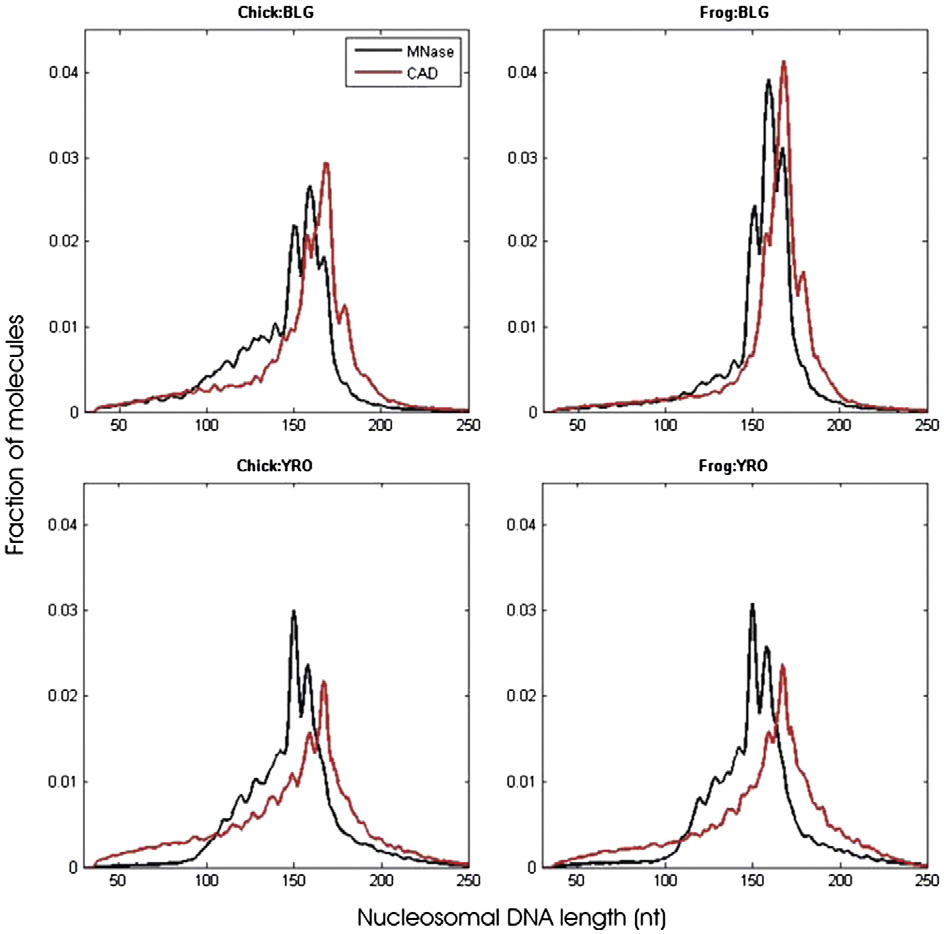
Size distributions of the histone octamer binding sites. In the top two panels, the distributions obtained from the sequence reads of nucleosomal DNAs prepared with MNase (black) or CAD (red), derived from reconstitutes formed on BLG using either chicken (left) or frog (right) histones, are shown. The equivalent profiles for nucleosomal DNAs derived from YRO are shown in the bottom two panels.

The size distributions of the paired-end reads that aligned to the two sequences reveal the lengths of DNA protected by the histone octamer during nuclease digestion (Fig. 3). In all cases, the distributions fall largely within the expected size range for mononucleosomal DNA. Notably, in all samples, the DNA lengths are distinctly quantized. For example, in the MNase-derived monomer DNAs, clear peaks are observed at ~ 149, ~ 159, and ~ 168 nucleotides. The main peak in the MNase distributions tends to be at 159 nucleotides for nucleosomes derived from the BLG DNA, whereas for YRO, the major species contains 149 nucleotides. As MNase produces two-nucleotide-long 5′ end extensions at the site of cleavage,^24^ the 149-nucleotide read length is consistent with a substantial fraction of the nucleosome population being of core particle length (~ 147 bp) and much of the remainder falling into classes containing an extra ~ 10 or ~ 20 bp of DNA, as previously observed.^31^

Digests produced with CAD nuclease also give rise to quantized nucleosomal DNA lengths although the major peak in these digests is consistently at 168 bp, irrespective of the DNA source. It is notable that in the CAD digests, there is relatively little material of typical core particle length (147/149 bp). Although a small fraction of the nucleosomal DNA derived from the YRO sequence reconstituted with chicken histones shows a discernible peak at about 149 bp, there is little evidence of a corresponding peak in any of the other digests (Fig. 3).

**Fig. 4:**
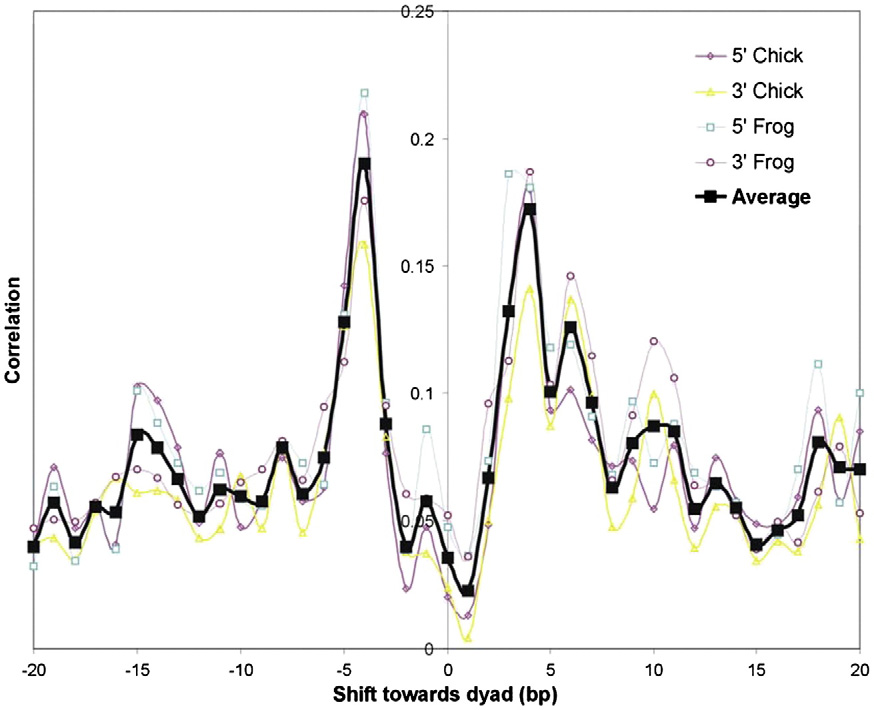
Cross-correlation analysis of sequence read location profiles for monomer DNAs derived from BLG reconstitutes after digestion with either MNase or CAD nuclease. Separate results are shown for a comparison of the upstream (5′) or downstream (3′) ends of corresponding data sets, for samples prepared by reconstitution with chicken or frog histones and for an average of all these sets.

**Fig. 5:**
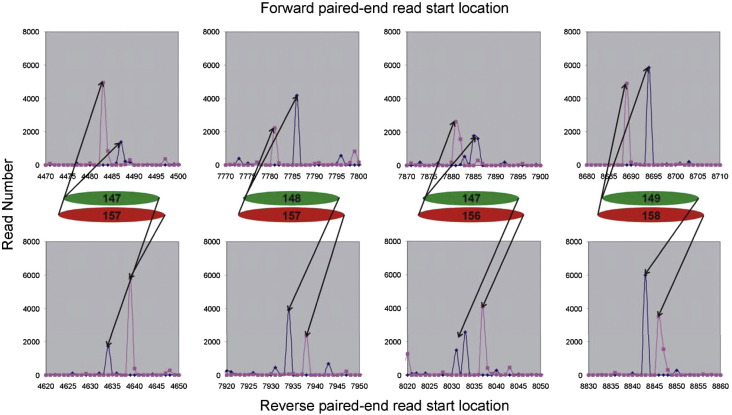
Schematic interpretation of the nucleosome structures indicated by the locations of the upstream and downstream ends of sequence reads derived from monomer DNAs produced by digestion of chromatin, formed by reconstitution of BLG with frog histones, by MNase (blue and green) or CAD nuclease (purple and red).

Although the quantized lengths of the DNAs derived from MNase and CAD digestion of chromatin are equivalently sized, this does not correspond to cleavage of the DNA, by each enzyme, at the same positions relative to the nucleosomal structure. Cross-correlation of the locations of the MNase and CAD cleavage sites shows that they tend to be shifted with respect to each other by about 4–5 bp (Fig. 4). Thus, CAD nuclease appears to cut at a site 4–5 bp further from the core particle boundary than does MNase. Numerous examples confirming this interpretation can be identified from visual inspection of the corresponding maps of paired-end sequence read locations derived from MNase- and CAD-digested chromatins. From the results shown in Fig. 5, four histone octamer sites can be identified. For the MNase data, each site contains 147, 148, or 149 bp of DNA, whereas for the CAD data, the same sites contain 157 or 158 bp of DNA due to being about 5 bp longer at both the upstream and downstream ends of the binding site. As CAD digestion of chromatin produces a “core particle” containing about 157 bp of DNA, this explains the lack of typical (~ 147 bp) core particle length products in the CAD digests (Fig. 3).

**Fig. 6:**
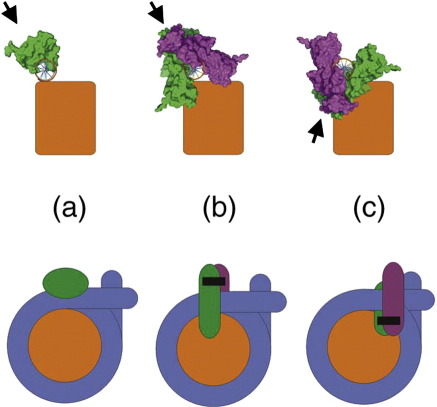
Schematic representation, from two perspectives, of the binding of MNase (a) and CAD nuclease (b and c) to nucleosomal DNA during cleavage. The core histone octamer is represented as an orange cylinder and, in the lower panel, the nucleosomal DNA is coloured blue. The direction of cleavage into the minor groove of the DNA is indicated by the arrows. In (c), the dimeric CAD nuclease has been rotated clockwise by 140° relative to its position in (b), corresponding to a translation of about 4 bp.

A simplistic consideration of how MNase and CAD might interact with the nucleosome structure during cleavage provides a possible explanation for the above behaviour (Fig. 6). The relatively small, monomeric molecule of MNase can easily access the outward-facing, minor groove cleavage site at the boundary of the canonical core particle^9^ (Fig. 6a), whereas the larger, dimeric CAD complex will not be able to bind and cut at the same site due to the steric hindrance between the inward-facing “blades” of the enzyme and the histone octamer (and adjacent gyres of DNA) (Fig. 6b). However, the relative shift of the CAD enzyme would be expected to reduce this steric hindrance in two ways: Firstly, the translation of the enzyme 4–5 bp along the axis of the DNA at the termini of the core particle will effectively move it away from the core histone octamer. Secondly, and in the present context probably more importantly, the blades of the enzyme will now rotate to point away from the histone octamer (Fig. 6c). This  5-bp rotational shift of the CAD site relative to the MNase site is also maintained at cleavage locations further from the core structure. Thus, CAD nucleosomes containing ~ 167 bp of DNA are formed by cleavage at a point ~ 10 bp either upstream or downstream of the CAD sites that give rise to the  157-bp core particle (Supplementary Fig. 3).

### Generation and properties of histone octamer positioning maps

We have used two methods to represent the histone octamer binding sites (nucleosome positions) identified on each of the DNAs. Firstly, the data are presented simply in terms of the coverage attained through sequencing. Secondly, we have also employed a previously developed procedure^31^ to identify positioning site dyads.

**Fig. 7:**
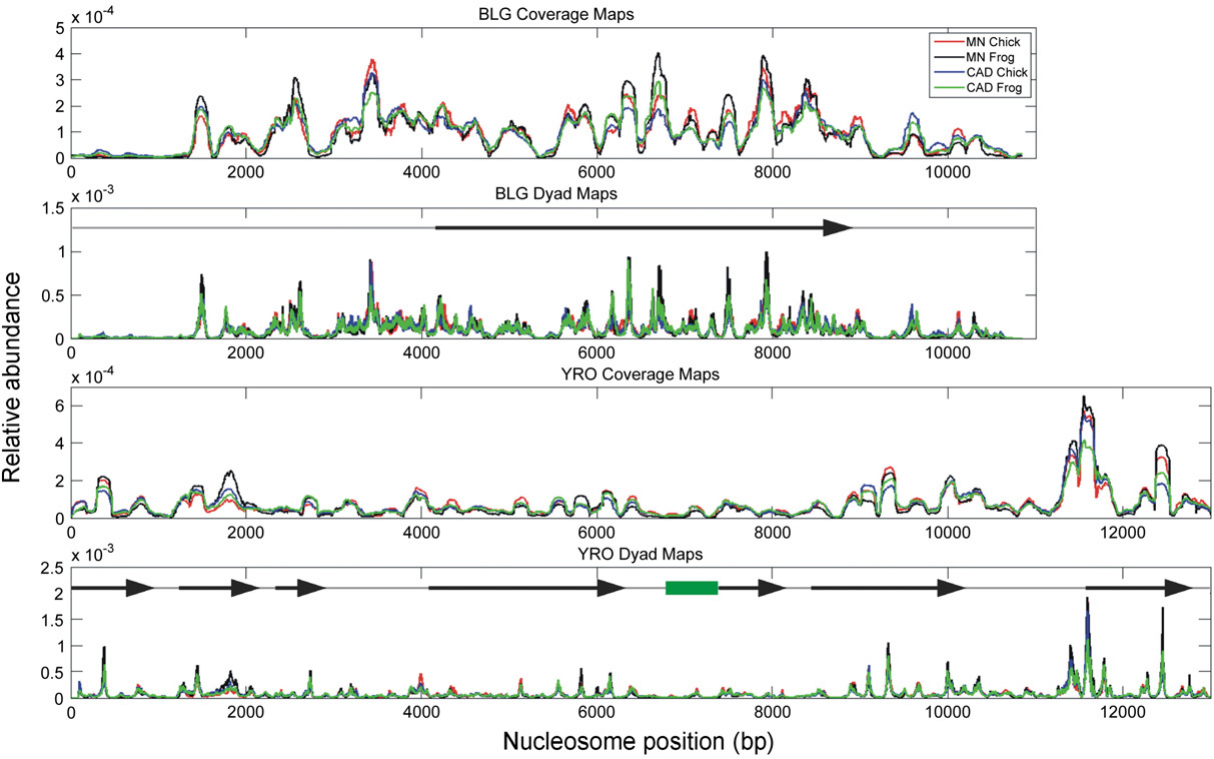
Core histone octamer positioning on genomic DNA sequences. The histone octamer binding sites identified on BLG (top two panels) and YRO (bottom two panels) are presented in terms of the type of core histone used for chromatin reconstitution and the type of nuclease used to digest the chromatin. The maps were generated from paired-end sequencing reads of nucleosomal DNAs and are presented in terms of either sequence coverage or positioning site (binding site) dyads. The maps were normalised, for each DNA sequence, not including the vector sequence, to the total signal intensity. Schematic representations of the gene structures (transcribed sequences) within each of the genomic regions for each plasmid are shown (arrows) and the location of the replication origin (YRO) is identified by the green rectangle.

Histone octamer positioning maps for the BLG gene sequence reconstituted with chicken or frog histones and digested with either MNase or CAD nuclease are shown as coverage and positioning site dyad maps in the upper two panels of Fig. 7. The corresponding maps for the YRO sequence are shown in the bottom two panels. The maps have been normalised with respect to the total number of aligned sequence reads obtained from each sample and have been corrected for the relative molar amounts of each plasmid DNA. Consequently, for each of the separate sequences, the maps can be directly compared in terms of the relative intensity and distribution of histone octamer binding sites. A separate set of maps in which only the corresponding MNase and CAD profiles are compared is provided in Supplementary Fig. 4.

In comparing the character of the histone octamer-binding maps, that of BLG stands out from YRO in terms of the abundance of positioning sites that are frequently occupied (high-affinity sites). Generally, the yeast sequence displays a relatively low number of strong positioning sites, although the most abundantly occupied positioning site identified in our analysis (on both BLG and YRO) is found towards the 3′ end of the yeast sequence (11,590 bp). Recalling that the two DNAs were reconstituted, digested, and gel purified as a mixture, the differences between the affinity profiles can only be attributed to sequence composition, and its influence during the competitive conditions used for reconstitution.

A visual comparison of the histone octamer positioning maps (Fig. 7 and Supplementary Fig. 4) indicates a high degree of similarity on each DNA irrespective of histone type used for reconstitution or the enzyme type used for the preparation of nucleosomes. Even at higher resolution, when mapping positioning site dyads, the profiles are strikingly similar. Generally, for both DNAs, it seems clear that (i) chicken and frog histones bind with equivalent affinity to the same spectra of positioning sites during reconstitution and that (ii) the digestion of the resulting chromatin with either MNase or CAD nuclease gives rise to equivalent populations of DNA binding sites.

Although the MNase and CAD positioning maps are in general very similar, there are a few particular instances where quantitative differences in nucleosome occupancy appear notable. For example, there is a binding site on the BLG map centred on bp 9700 where the occupancy on the MNase map is less than that on the CAD map (Fig. 7 and Supplementary Fig. 4). This observation might be consistent with the proposal that the use of MNase leads to an underrepresentation at this binding site. However, there is a another site on the YRO map centred on bp 12,500 where the opposite conclusion is reached because the occupancy on the MNase map is clearly greater than that on the CAD map (Fig. 7 and Supplementary Fig. 4). It is unclear why these differences arise in these particular instances although cleavage specificity of the enzymes or differences in digestion conditions could be contributing factors.

To further compare the various histone octamer binding site data sets, we have employed scatter plots. For this purpose, we converted the occupancy profiles to indicate the relative free energy (Δ*G*^0^) of association of the histones with the DNA using the following equation:

$$\Delta G_{i}^{0}=-RT\text{ln}\left(I_{i}/I_{\text{ref}}\right)$$

where *R* is the molar gas constant, *T* is the temperature in Kelvin, *I*_*i*_ denotes the level of binding site occupancy of positioning site *i*, and *I*_ref_ represents the binding site occupancy of the reference site, which we chose to be the highest-affinity site in the data sets (11,590 bp on the YRO sequence; Fig. 7).

**Fig. 8:**
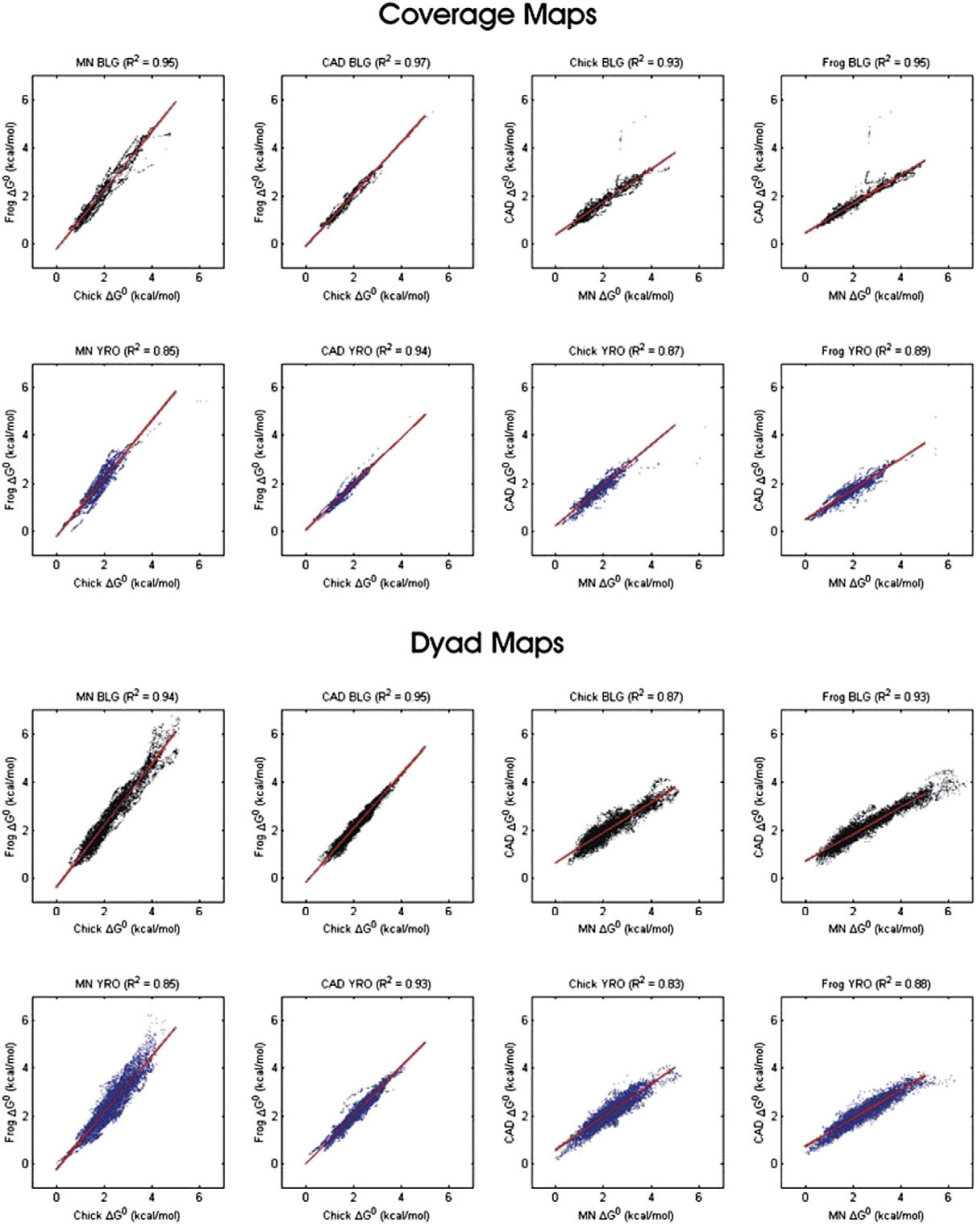
Relationships between histone octamer binding site affinity maps. Scatter plots comparing the relative free energy (Δ*G*^0^) of positioning sites measured on BLG (black symbols) or YRO (blue symbols) are shown. Both coverage and positioning site dyad maps were analysed. Various comparisons between data derived from reconstitutes prepared with different types of core histone and for monomer DNAs prepared with different nucleases are presented. For each scatter plot, the *R*^2^ value, derived from linear regression of the data (red line), is presented.

**Fig. 9:**
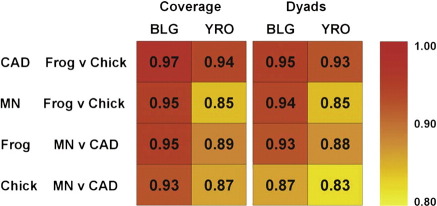
Summary of relationships between histone octamer binding site affinity maps. The *R*^2^ values derived from linear regression analysis of the scatter plots of the histone octamer positioning profiles (Fig. 8) are presented in a colour-coded format.

The resulting scatter plots, where, for each type of DNA, samples are compared in terms of histone type used for reconstitution and separately in terms of nuclease type used for chromatin digestion, are shown in Fig. 8. The *R*^2^ values derived from linear regression analysis of the scatter plots are summarised in Fig. 9. The latter values range from a high of 0.97 (a comparison of the frog and chicken coverage maps on BLG DNA produced with CAD nuclease) to a low of 0.83 (a comparison of the CAD and MNase positioning site dyad maps on YRO DNA reconstituted with chicken histones). Correlations between the positioning site dyad maps are generally slightly lower than those between the coverage maps. However, the average *R*^2^ value for all the BLG data sets only drops from 0.95 to 0.92, and that for YRO drops from 0.88 to 0.87. Thus, the striking correspondence is retained when the analysis is carried out at high resolution.

Overall, this analysis demonstrates an exceptionally strong relationship between the nucleosome positioning maps produced on different DNAs, with separate types of core histones and with different types of nucleases, and indicates that only small changes can be attributed to any of these variables. Consequently, our results do not suggest that, under our conditions, the use of MNase substantially biases the identification and characterisation of nucleosome positioning sites.

## Discussion

The primary purpose of this study was to assess the extent to which the use of MNase could bias nucleosome positioning information. Potential biases have been suggested^4–6^ for two main reasons. Firstly, the nuclease displays a strong preference to cut DNA at sites containing an A/T dinucleotide at the point of cleavage.^7–9^ Secondly, because the enzyme is relatively small compared to the nucleosome (Fig. 1), it is supposed that it can access and cut at sites comprising outward-facing, minor grooves of the DNA within the core particle structure itself. In combination, these two properties raise the prospect that MNase, during extensive digestion of chromatin, can selectively degrade and consequently remove a fraction of the nucleosomal DNAs of particular sequence compositions, thereby biasing the nucleosome positioning information derived from analyses of the remaining, resistant nucleosomal DNA fraction.

It is important to stress that the critical feature of the above scenario concerns the ability of the nuclease to make a double-stranded cut in the DNA wrapped around the histone octamer. MNase can make such cleavages under extensive digestion conditions.^9,11^ However, these tend to be located towards the periphery of the nucleosomal structure and are probably substantially dependent upon the transient unwrapping of the DNA from the histone octamer surface.^32–34^ There is in fact no evidence that double-stranded cleavage by MNase, at internal sites on the nucleosome, occurs with any substantial frequency when the DNA remains attached to the histone core, irrespective of the sequence composition of the outward-facing minor groove.

Like MNase, CAD displays a strong preference to cut the linker DNA between nucleosomes in chromatin.^23^ However, unlike MNase, there is little prospect that this latter enzyme can access, let alone cut, nucleosomal DNA bound to the histone octamer. The primary reason for this would appear to derive from the architecture of the active, dimeric CAD enzyme. As it is shaped like a pair of scissors (Fig. 1), there is an absolute requirement that the DNA substrate is not bound to a protein surface that would sterically restrict the approach of the enzyme. This point appears to be borne out by our data that indicate that in order to cleave the DNA at the boundary of the core particle, CAD nuclease rotates and translates along the helix, away from the nucleosome dyad, so that the blades of the enzyme can avoid steric restriction (Fig. 6c). As a result, a core particle produced by CAD nuclease contains DNA that is 8–10 bp longer than the equivalent MNase-produced particle. Furthermore, this relative translation between the cleavage sites of the two enzymes indicates that whereas MNase cuts in the outward-facing minor groove of the nucleosomal DNA, CAD nuclease cuts close to the inward-facing minor groove (or at a site in phase with it).

It follows that, in the context of the current study, there are three relevant differences between MNase and CAD nuclease. Although the characteristic manner in which these enzymes digest chromatin is dictated by nucleosome structure, the dissimilarity in enzyme architecture leads to the use of distinctly different sites of nucleosomal DNA cleavage. In addition, compared to the limited ability of MNase to cut at sites internal to the nucleosome, such behaviour is likely to be substantially reduced in the case of CAD nuclease. Finally, although both enzymes display sequence bias in terms of their preferred cleavage sites, they are quite distinct in this respect (Fig. 2). Given these notable differences, a comparison between nucleosomal DNA populations produced with either MNase or CAD nuclease should be eminently appropriate to assess potential bias in nucleosome positioning resulting from the use of MNase.

Our nucleosome positioning analyses suggest that there is very little difference between the nucleosomal DNA populations derived from reconstituted chromatin after digestion with either MNase or CAD nuclease. These observations are not consistent with the use of MNase introducing a systematic and consistent bias to the composition of nucleosomal DNA populations and a subsequent bias to the nucleosome positioning information derived from these populations. If this conclusion were to apply generally, it would follow that the high correspondence between MNase-generated maps of naked DNA and nucleosome positioning maps obtained from native or reconstituted chromatins^4,5^ is not simply coincidental but reflects a pattern of sequence-based, genomic organisation that may be fundamentally linked to the biological requirements and consequences of packaging DNA into chromatin and highlights the essential role of nucleosome positioning in this process. However, further studies, particularly with native chromatins, will be required to substantiate these conclusions.

## Methods

### DNA and histones

Two plasmid DNAs were employed: pBLG (BLG) comprised 10,841 bp of ovine DNA containing the BLG gene and 2020 bp of plasmid vector;^31^ p13 (YRO) comprised 13,626 bp of *S. cerevisiae* DNA (chrXIV:243,179–256,806 [(SacCer_Apr2011/sacCer3) assembly]) containing a late-firing replication origin [ARS1413 (~ 250,600–251,220)] and 6683 bp of plasmid vector.^31^ The plasmid DNAs were propagated in a *dam*, *dcm* bacterial strain and consequently would have been subject to bacterial methylation. Neither DNA was linearised prior to reconstitution. Chicken erythrocyte core histones were prepared as previously described.^24,25^ Recombinant *Xenopus laevis* histones^35,36^ were purified and refolded, and octamers were isolated.^36^

### DFF/CAD nuclease preparation

Cloned mouse DFF45 and DFF40 subunits were co-expressed and purified as previously described.^37^ The protein was stored in 100 mM KCl, 20 mM Tris–HCl, pH 8.0, 0.2 mM ethylenediaminetetraacetic acid, 2 mM DTT, and 10% glycerol. Enzymatic activity was empirically measured by digesting naked DNA. The DFF/CAD nuclease was activated by digestion with tobacco etch virus enzyme (Invitrogen) at 30 °C immediately before use.

### Nucleosomal DNA preparation

A mixture of equal weights of linearised plasmids containing BLG and YRO were reconstituted with core histones by salt gradient dialysis.^24,25^ In independent experiments, chicken and frog histones were used to prepare reconstitutes. Nucleosomal DNA was prepared from these reconstitutes using MNase as previously described.^25^ Briefly, 25 μg of reconstituted chromatin was digested with 3 U of MNase (Worthington) for 30 min on ice, followed by 3 min at 37 °C. For digestion with CAD/DFF nuclease, 25 μg of reconstituted chromatin was digested with activated enzyme at 37 °C for 60 min in 10 mM Tris–HCl, 16 mM KCl, 3 mM MgCl_2_, and 0.2 mM PMSF. The resulting ~  146-bp mononucleosome DNA fragments were purified after electrophoresis on 1.5% agarose gels.

### DNA sequencing

Illumina/Solexa paired-end sequencing was undertaken by The Gene Pool at Edinburgh University†. Preprocessing involved blunt-ending of nucleosomal DNA by filling-in, adapter ligation, and amplification by 18 cycles of PCR.

Sequencing data and associated metadata can be found at the EBI Sequence Read Archive (http://www.ebi.ac.uk/ena/data/view/ERP001171). Reference sequences for pBLG and pYRO are available at our web site (http://www.enps.bio.ed.ac.uk).

### Alignment of sequence reads to the reference sequence

Paired-end and single-end sequence reads were aligned to the reference sequence using Bowtie.^38^

### Generation of nucleosome positioning maps

Nucleosome positioning data are presented in two ways. Coverage maps reflect the occurrence of each nucleotide of the mapped DNA in the aligned sequence reads. Alternatively, maps depicting the dyads of histone octamer binding sites (positioning site dyads) have been generated essentially as previously described (Method 2).^39^ In this approach, a range of possible nucleosomal DNA lengths (all odd-numbered lengths from 121 to 191) are considered. Each 5′ read count is paired with the appropriate 3′ read count for the nucleosome length being considered, and the positioning site dyad established midway between these points. The amplitude of peak corresponding to a dyad is determined by the geometric mean (square root of the product) of the forward and reverse read counts. This method generates 36 maps, 1 for each nucleosome size being considered, which were summed for the final map. For comparison, all the above maps were normalised to the total occurrence of positioning sites within each map.

### Molecular graphics

Possible modes of interaction between MNase^20^ [Protein Data Bank (PDB) ID: 2SNS] or CAD^17^ (PDB ID: 1V0D) and DNA were analysed using PyMOL software.^40^

## Footnotes

† http://genepool.bio.ed.ac.uk/
