# Supplementary material for: Micrococcal Nuclease Does Not Substantially Bias Nucleosome Mapping
Source: J Mol Biol. 2012 Mar 30;417-135(3):152–64. doi: 10.1016/j.jmb.2012.01.043 (PMC3314939; doi:10.1016/j.jmb.2012.01.043)
Supplement: Supplementary file 1 — Supplementary data [file mmc1.pdf]

Supplementary Figure Legends

Supplementary Figure 1

Agarose gel (1.5%) electrophoresis of DNAs purified from reconstituted chromatins digested on a time course basis with micrococcal nuclease (A) or caspase activated DNase (B). Digestion with MNase was on ice for the times indicated (min), followed by 3 min at 37°C. Digestion with CAD nuclease (at two separate enzyme:chromatin ratios) was at 37ºC for the times indicated (min). The resulting purified ~146 bp mononucleosome DNAs, were re-analysed on a 1.5 % agarose gel (C).

Supplementary Figure 2

Sequence properties of the cleavage sites for MNase and CAD nuclease. The occurrence of each nucleotide, both 5′ and 3′ of the cleavage point (position 0), for binding sites identified with MNase (left panels) or CAD nuclease (right panels) on BLG (top panels) and YRO (bottom panels) are shown. These data are an average of all sites identified from nucleosomal DNAs prepared from chromatins reconstituted with either frog or chicken histones.

Supplementary Figure 3

Schematic interpretation of the nucleosome structures indicated by the locations of the upstream and downstream ends of sequence reads derived from monomer DNAs produced by digestion of frog histone-reconstituted chromatin with CAD nuclease. The data indicate the relationship between “core particles” (blue and green) and nucleosomes containing an additional 10 bp extension of DNA (purple and red).

Supplementary Figure 4

Core histone octamer positioning on genomic DNA sequences. The histone octamer binding sites identified on BLG (a-d) and YRO (e-h), reconstituted with chicken (a,c,e,g) or frog (b,d,f,h) histones and digested with MNase (black) or CAD nuclease (red) are presented in terms of sequence coverage (a,b,e,f) or nucleosome dyads (c,d,g,h). The maps were generated from paired-end sequencing reads of nucleosomal DNAs and were normalised, for each DNA sequence, not including the vector sequence, to the total signal intensity.

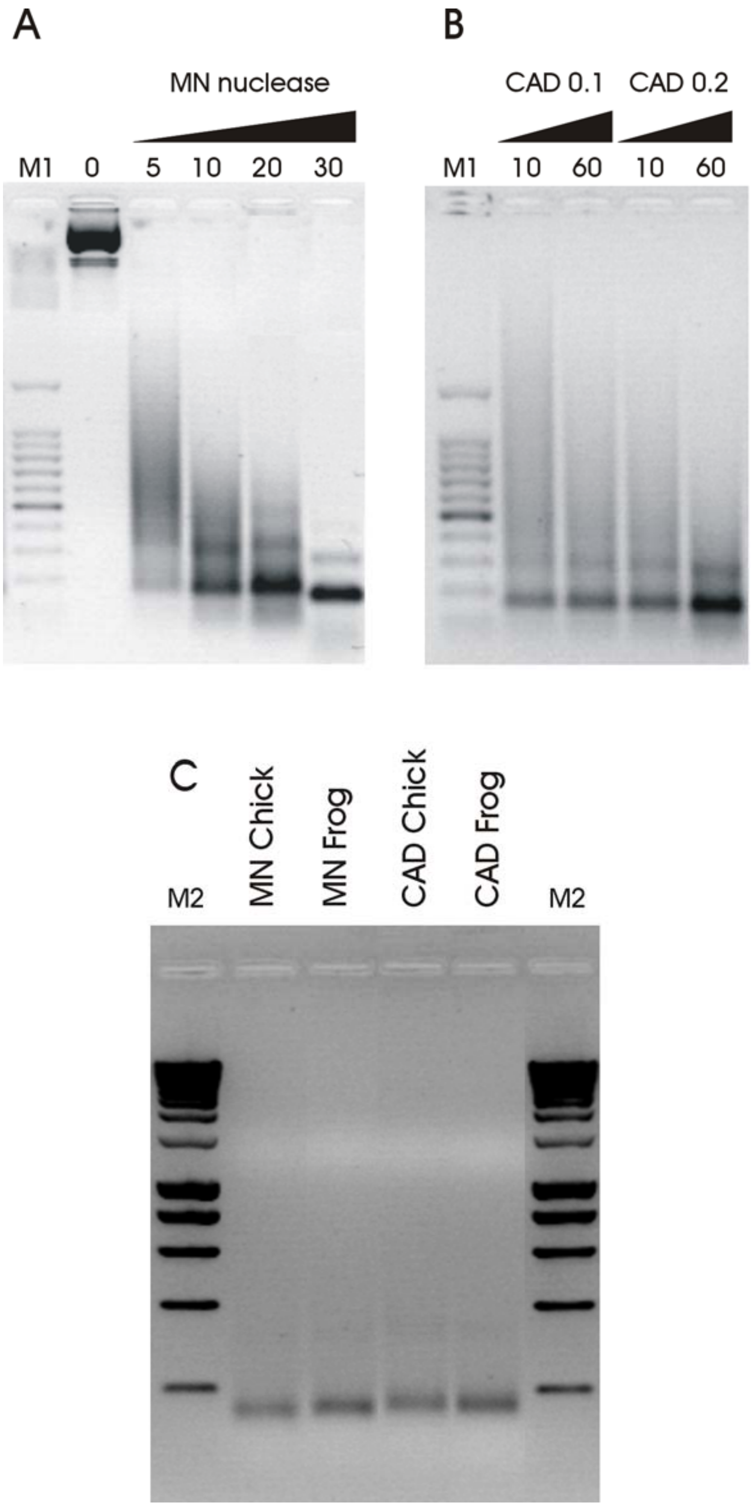

Supplementary Figure 1
Allan et al., 2012

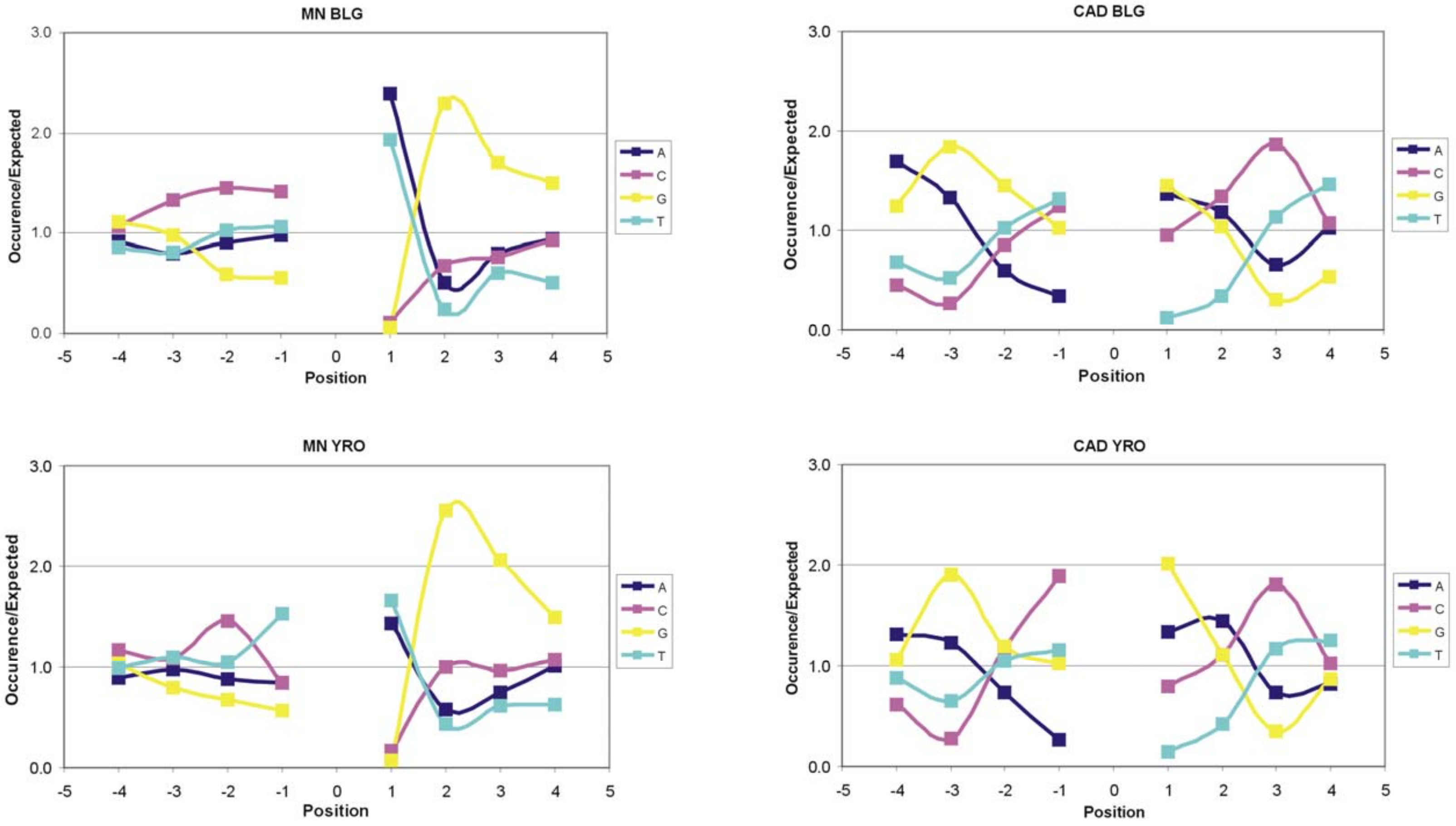

Supplementary Figure 2
Allan et al., 2012

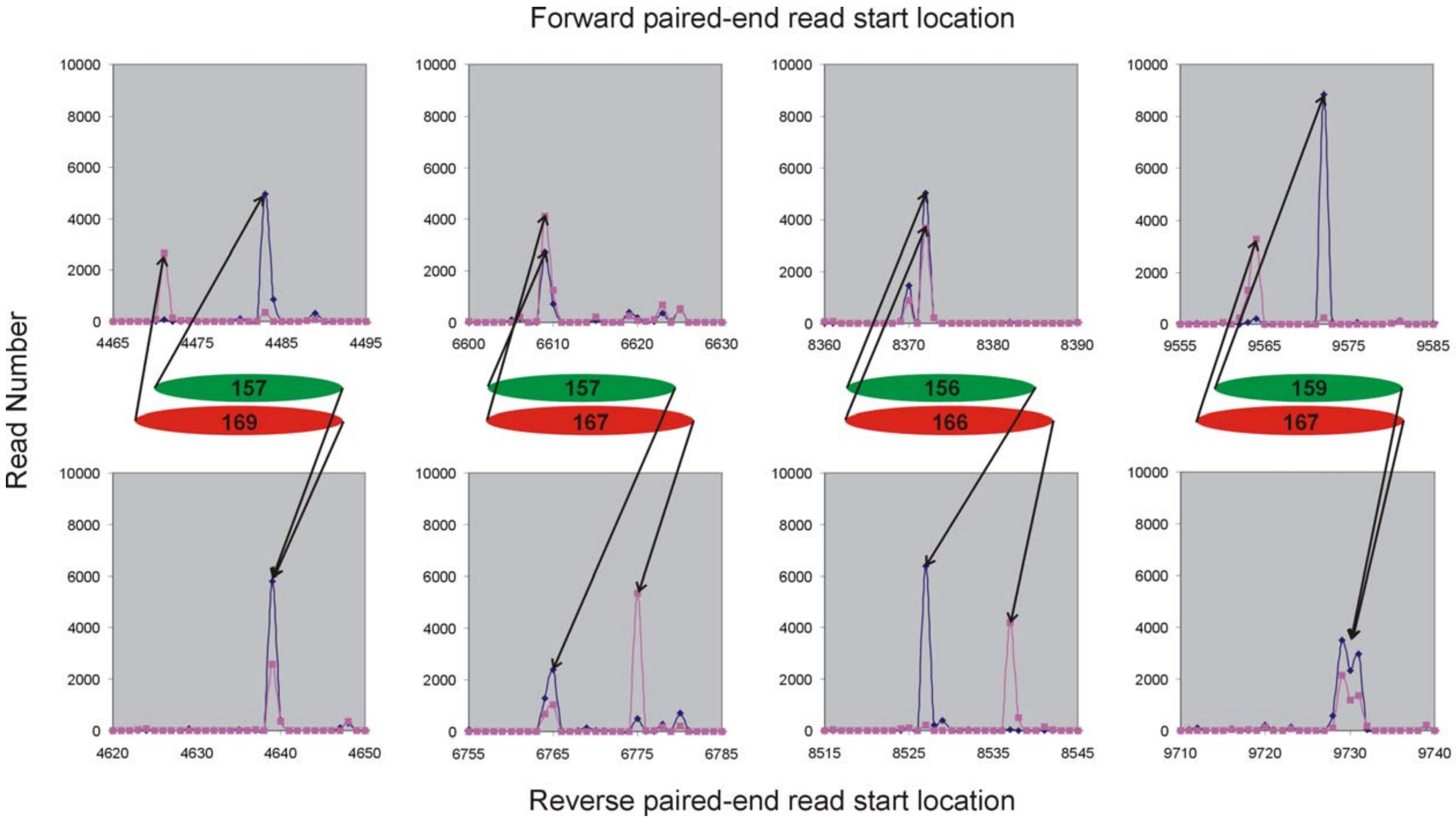

Supplementary Figure 3
Allan et al., 2012

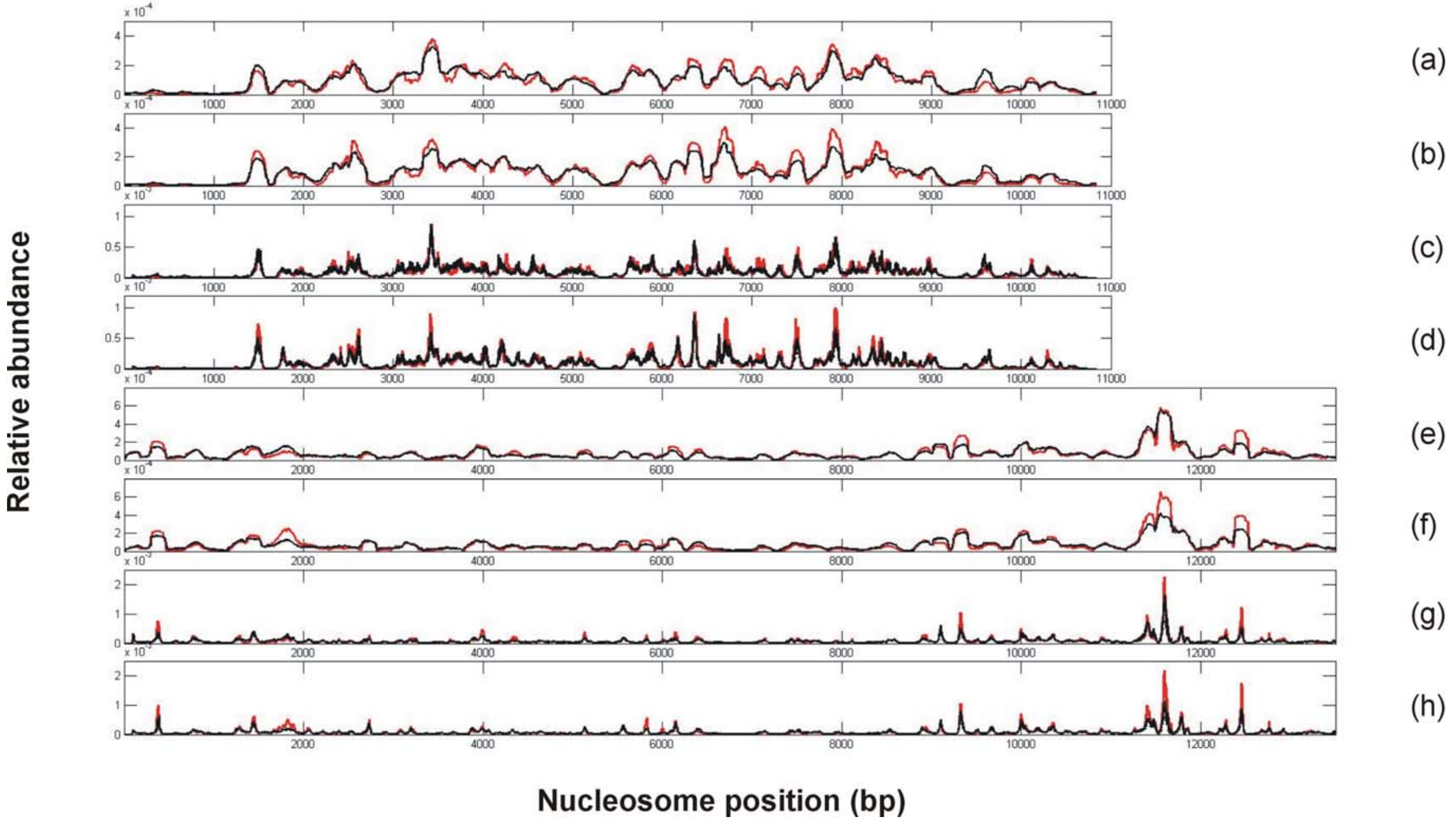

Supplementary Figure 4
Allan et al., 2012
